# Economic Sanctions Affecting Household Food and Nutrition Security and Policies to Cope With Them: A Systematic Review

**DOI:** 10.34172/ijhpm.2023.7362

**Published:** 2023-08-05

**Authors:** Fatemeh Mohammadi-Nasrabadi, Delaram Ghodsi, Arezoo Haghighian-Roudsari, Fatemeh Esfarjani, Mohammad-Reza Khoshfetrat, Zeinab Houshialsadat, Maryam Mohammadi-Nasrabadi, Ghasem Fadavi, Reza Majdzadeh

**Affiliations:** ^1^Research Department of Food and Nutrition Policy and Planning, National Nutrition and Food Technology Research Institute, Faculty of Nutrition Sciences and Food Technology, Shahid Beheshti University of Medical Sciences, Tehran, Iran; ^2^Department of Nutrition Research, National Nutrition and Food Technology Research Institute, Faculty of Nutrition Sciences and Food Technology, Shahid Beheshti University of Medical Sciences, Tehran, Iran; ^3^Department of Community Nutrition, National Nutrition and Food Technology Research Institute, Faculty of Nutrition Sciences and Food Technology, Shahid Beheshti University of Medical Sciences, Tehran, Iran; ^4^School of Public Health, Physiotherapy and Sports Sciences, University College Dublin, Dublin, Ireland; ^5^Department of Health Education and Promotion, School of Public Health, Tehran University of Medical Sciences, Tehran, Iran; ^6^Food, Halal and Agricultural Products Research Group, Research Center of Food Technology and Agricultural Products, Standard Research Institute, Karaj, Iran; ^7^Interdisciplinary Research and Practice Division, School of Health and Social Care, University of Essex, Colchester, UK

**Keywords:** Economic Sanctions, Food Security, Policy, Food System, Systematic Review

## Abstract

**Background:** This review was conducted to identify the impact of economic sanctions on household food and nutrition security and policies to cope with them in countries exposed to sanctions.

**Methods:** The Preferred Reporting Items for Systematic Reviews and Meta-Analyses (PRISMA) guidelines 2020 were used to identify, select, appraise, and synthesize studies. Electronic databases in addition to Persian ones have been systematically searched for all related documents published until March 2022. Exclusion criteria were: lack of data related to food insecurity in countries subject to sanction and very low quality of the article. The quality of included studies was assessed using the Joanna Briggs Institute (JBI) Critical Appraisal checklists. The results were presented as qualitative and quantitative syntheses.

**Results:** Of 1428 identified studies, 36 publications remained in the review, which belong to Iran (n=8), Cuba (n=8), Russia (n=7), Iraq (n=7), and Haiti (n=6), respectively. Declining gross domestic product (GDP), devaluation of the national currency, and the quality of food, increase in inflation, unemployment, and consumer prices, infant and under 5 years mortality, energy, and protein deficiency, and the poverty rate were reported as sanction consequences. The most important strategies to improve food security were the humanitarian assistance provided by the international community (Haiti), equity and priority for vulnerable groups mainly by expanding the healthcare system (Cuba), adopting a food ration system in the oil-for-food program, and fixing the price of food baskets (Iraq), import substitution and self-sufficiency (Russia), support domestic production, direct and indirect support and compensation packages for vulnerable households (the approach of resistance economy in Iran).

**Conclusion:** Due to the heterogeneity of studies, meta-analysis was not possible. Since inadequate physical and economic food access caused by sanctions affects especially disadvantaged and vulnerable groups, planning to improve food security and providing support packages for these groups seems necessary.

## Background

 Sanctions are interruptions of a country’s communications, diplomatic, and/or economic relations and are considered to be a positive tool to facilitate the resolution of conflict in a less violent manner. However, economic sanctions that affect trade and finance can cause severe economic destabilization with grave impacts on the livelihoods and well-being of populations in affected countries.^1^ Economic sanctions are imposed by governments or the United Nations Security Council against individuals, companies, or countries. Iran and Iraq have experienced targeted economic sanctions (excluding food and medicines) by both the United Nations Security Council and the United States; whereas sanctions on Cuba by the United States (Unilateral) are comprehensive (including food, medicines, and medical equipment) and have extraterritorial components which effectively make it difficult for other countries to trade with Cuba. However, there is ample evidence of the adverse effects of any kind of economic sanctions on the welfare, health, and food security of civilians.^2^ It may appear as trade sanctions in the form of embargoes, seizures and/or boycotts, and interruption of financial and investment flows vis-à-vis a specific country. Recently, new forms of coercion have been emerging through asset freezing, asset control, and travel bans to influence persons who are perceived to have political influence.^3^

 Many of the sanctions imposed on countries around the world are aimed at weakening their economies, resulting in increased poverty, reduced food security, and public welfare. Although sanction objectives are rarely met, these sanctions nevertheless lead to a humanitarian disaster.^4,5^ Sanctions can influence all dimensions of food security: Availability would be limited by preventing civilians from acquiring related essentials such as farm implements, fertilizers, transportation equipment, spare parts, pharmaceuticals, or chemicals for sanitation. The Weakening of critical infrastructure including roads, ports, power plants, transformer stations, hospitals, factories, warehouses, and water and sanitation plants will disrupt the process, distribution, and access to food and may lead to the spread of diseases that make it difficult to absorb nutrients (utilization). A collapse in the education system, on the other hand, can worsen unemployment for years to come, in turn having an impact on access to adequate food.^5^

 This systematic review was conducted to identify the impact of economic sanctions on household food and nutrition security and policies to cope with them in exposed countries in the framework of sustainable food and nutrition security system. The practical aim was to provide solutions to prevent food insecurity in vulnerable households in the face of new sanctions against Iran.

## Materials and Methods

 The experiences of all exposed countries to economic sanctions and the experience of Iran with the previous round of sanctions, in particular from the escalation of sanctions in 2011 to the time of the Joint Comprehensive Plan of Action in 2015, were systematically reviewed. The Preferred Reporting Items for Systematic Reviews and Meta-Analyses (PRISMA) guidelines 2020 were used to identify, select, appraise, synthesize, and report studies in the present systematic review to prepare a transparent, complete, and accurate account of why the review was conducted, how it was done and what the results were.^6^ The study was registered in PROSPERO with the Number CRD42020191028.

###  Literature Search

 Electronic databases (Embase, PubMed, Scopus, Science Direct, Web of Science, JSTOR, and Google Scholar) in addition to Persian ones (SID, Magiran, IranDoc, and Noor) have been searched for all related documents published until March 1, 2022. There was no time limit for the start date of the search. Keywords relevant to sanction (economic sanction, embargo, or monetary sanction) and food and nutrition (food*, nutrition, nutrient*, “food secur*,” and “food insecur*”) were used. Moreover, some reference lists of identified studies, related projects, congresses abstract, dissertations, and relevant reviews were searched as gray literature to find more likely all eligible studies. According to the nature of the research topic, some sources eg, reports, preprints, working papers, and statements, produced by government departments and agencies, civil society or non-governmental organizations especially organizations providing humanitarian aid, academic centers and departments, private companies, and consultants were searched, too. There was no limitation on the time of papers. The search strategy of the review is presented in Supplementary file 1.

###  Article Screening, Inclusion, and Exclusion Criteria 

 Initial screening was performed using the title and abstract and then, full texts were downloaded if needed. After searching for related articles, they were selected and reviewed by the project executives to remove irrelevant items. All relevant results were extracted from cross-sectional, retrospective, surveys, cohort, before-after, interventional, and qualitative studies. There was no limitation for the target groups in terms of age and gender and language of published studies. Book chapters and available conference proceedings were also considered for more access to relevant data. Exclusion criteria were: lack of data related to food insecurity in countries subject to sanction and very low quality of the article. Duplicate citations and non-accessible old publications were excluded, too.

###  Data Extraction and Quality Assessment 

 Two reviewers (FM-N and DG) conducted systematic processes of literature searches, quality assessment, and data extraction of eligible papers independently and potential conflicts were resolved through discussion. In cases of disagreement, help was sought from a third person (AH-R). Data extracted from the selected studies included author’s name(s), year of publication, imposed countries, methodological characteristics (study design, sample size, and sampling method), sanction type and duration, the impact of sanctions on different dimensions of food security including availability, access, utilization, stability, policies, and programs to cope with them. Some related factors to the success of the programs and policies such as context and process were reviewed, too.

 The quality of included studies was assessed using the Joanna Briggs Institute (JBI) Critical Appraisal checklists for prevalence studies, quasi-experimental (non-randomized experimental) studies, and qualitative evidence. Each study can receive a high (H), medium (M), or low (L) quality rating.^7^

###  Statistical Analysis

 The main strategy in the analysis was data synthesis. The included studies found in each country were more than 4 studies; however, a meta-analysis of findings was not possible because of their heterogeneity in terms of the context of the society, studied dimensions of food security, policies to cope, and quality of the study. Therefore, the results were presented as a combination of qualitative and quantitative synthesis. In other words, the findings on the effects of the embargo on the food security of the countries and the strategies used to cope with them were reported from both quantitative and qualitative studies.

## Results

 A total of 1428 studies were identified through a search of databases and gray literature on the subject of food security in sanctions. After excluding 861 duplicate and unrelated papers, we found 240 studies that assessed the status of food security in countries exposed to sanctions. After the full-text review, 204 were completely excluded because of duplication of data, not having required data, and the very low quality of the article. Of the remaining 36 publications in the review, most papers (n = 36) belonged to Iran (n = 8), followed by Cuba (n = 8), Russia (n = 7), Iraq (n = 7), and Haiti (n = 6), respectively. Figure S1 (Supplementary file 2) shows the process of searching for and selecting appropriate articles in this systematic review.

 Most of the studies were based on the existing routine data gathered in the imposed countries. Only three studies reported findings from interviews with experts and informants (1 in Iraq and 2 in Cuba) and another one in Iraq conducted document analysis. Review findings on the impact of economic sanctions on food and nutrition security and coping strategies against sanctions based on the imposed countries are presented as follows in Tables 1-5.

**Table 1:** Impact of Economic Sanctions on Food and Nutrition Security and Policies to Cope With Them in Haiti Based on Included Studies in the Review

**Author, Date**	**Sanction Type/Years**	**Study Design**	**Population, Sampling, Sample Size**	**Impact of Sanctions on Food Security**				**Strategies to Cope**	**Quality of the Study**
				**Availability**	**Access**	**Utilization**	**Stability**		
Chelala, 1994^8^	United Nations sanctions	Results from 3 national surveys	Haitian population	-	The embargo on fuel has limited most people's access to healthcare - No fuel to provide proper services to vulnerable populations in the remote regions.	Infant mortality has greatly increased. 60% of children aged below 5 are estimated to be malnourished, and 3% of them have severe malnutrition.	-	Organize humanitarian aid under a single leadership. No improvements will be sustainable unless the political crisis would be over.	Medium
Mulder-Sibanda, 1998^9^	International sanctions which became extremely stringent in 1993-1994	Examination of the nutritional status	Haitian children	Decline in GDP.	-	9% increase in mortality among young children corresponding to the trends in malnutrition. The prevalence of wasting almost doubled. Physical access to health services worsened.	-		Medium
Garfield, 1999^1^	Trade, except humanitarian goods/1991-1994 by the US and the OAS	Review	Data available from the Haiti population	Agricultural production declined 20%, GNP declined by 30%.	The price of staple foods increased fivefold; Industry employment declined 8%; The value of the national currency plummeted and hyperinflation occurred.	Increase in low birth weight, underweight, acute and chronic malnutrition in children and adults, infant mortality.	The export of mangoes, on which many poor depended, was halted.	Humanitarian assistance by the United Nations (mainly the US); feeding programs, immunization supplies, and a humanitarian fuel program.	High
Gibbons and Garfield, 1999^10^	Economic sanctions	Review data	National surveillance systems, service statistics from humanitarian organizations, and special studies on employment, income, nutrition, and mortality	Per capita gross national product declined by $ 120, or 30%.	The price of rice increased 137% and the price of corn increased 184%. Declining incomes forced people to reduce household expenditures and the quality of food were declined.	The mortality of children 1-4 years of age rose from 56 per 1000 to 61 per 1000 (measles epidemic) and infant mortality declined by 38%. Low birth weight increased from 10% to 15% due to maternal malnutrition.	Prevention of the export of $15 million of coffee and cocoa, $12 million of mangoes, and $14 million of essential oils, while it accelerated deforestation and erosion.	The international community provided Haiti with humanitarian assistance totaling an estimated $250 million, or $35 per capita.	High
Farmer et al, 2003^11^	Economic sanctions and embargoes	Review existing data	A population-based sample.	Crops were not planted because many parents, especially fathers, were in hiding.	A rise in the number of trauma cases attributable in large part to road accidents (patients have to travel long distances to receive care).	A reduction in the availability of potable water (from 53% to 35%); Child mortality doubled; measles outbreak as a consequence of deterioration of the public health infrastructure and shortages of food, medicine, and other supplies; 22% of child deaths were associated with severe malnutrition or kwashiorkor.	-	The USA, other “donor nations,” and multilateral organizations promised US$ 500 million over 2-3 years in development aid to rebuild Haiti’s battered health, education, and sanitation infrastructure.	Medium
Reid et al, 2007^12^	International embargo/1991-1994	Data systematically collected from 1989 to 1996	Longitudinal anthropometric records on 1593 children, 24 months old or younger.	-	-	The incidence of childhood mortality and severe malnutrition were higher during the period of the embargo than in the periods before and after the embargo.	-	-	Medium

Abbreviations: GDP, gross domestic product; GNP, gross national product; OAS, Organization of American States.

**Table 2:** Impact of Economic Sanctions on Food and Nutrition Security and Policies to Cope With Them in Iraq Based on Included Studies in the Review

**Author, Date**	**Sanction Type/Years**	**Study Design**	**Population, Sampling, Sample Size**	**Impact of Sanctions on Food Security**				**Strategies to Cope**	**Quality of the Study**
				**Availability**	**Access**	**Utilization**	**Stability**		
Drèze and Gazdar, 1992^13^	Embargo on all imports to and exports except for medical purposes, and, in humanitarian circumstances, foodstuffs/1990, following the invasion of Kuwait	Quantitative and qualitative	Data was collected through household surveys, and a large number of interviews with household members, factory managers, United Nations personnel, relief workers, government official, etc.	Iraq’s public distribution system is remarkably comprehensive, equitable, efficient, and reliable.	The level of employment has more or less stagnated, money wages have also roughly stagnated, and prices have increased very sharply leading to short-term local shortages and speculation, quantity constraints on the supply of imported goods due to sanctions, and depreciation of the unofficial exchange rate of the Iraqi dinar.			To store adequate amounts of food (either imported from abroad or procured within the country), distribute “ration cards” to the population, and supply the agents. Food is supplied to the agents every month according to the number of “coupons” which they can produce.	High
Garfield, 1999^14^	The economic sanctions on all items except medicines/1990	Review the information from 22 field studies	Iraqi children under five years of age.	A rapid decline in per capita product.	A rapid decline in the GDP and its most important component, export earnings from the petroleum sector led to a rapid rise in inflation and food prices for goods not purchased via ration.	Most Iraqi children survive under the social, economic, and political crises of the 1990s in Iraq but experience profound limitations on their health and wellbeing. Access to piped water is high but its quality has declined.		Implementing the oil for food program.	High
Garfield, 1999^1^	All items imported to Iraq, except Medicines/1990-1999	Review	Iraqi population.	Grain and meat production fell.	Purchasing power and educational achievement receded.	The energy, water, medical, and transportation infrastructure declined; Diarrhea and war-related mortality rose.			High
Popal, 2000^15^	NR	Health research	All Iraqi population.	3120 kcal energy vs. 1093 kcal (65.0% decrease); 62.5 g vs. 20.9 g Protein (64.7% decrease).	Widespread unemployment and shortage of hard currency led to the significant erosion of purchasing power of most families.	The failure in the importation of high quantities of medical supplies led to a neutral impact on the health services. Increasing all forms of malnutrition in children, unsafe drinking water, polluted environment + poor sewage.		Oil-for-food program led to improvements in the availability of food, drug, medical supplies, the memorandum of understanding between the United Nations and Iraq, the distribution of governmental food rations.	Medium
Koc et al, 2007^5^		-	-	Breakdown of food distribution led to countrywide food shortage, widespread malnourishment, and pre-famine conditions in some areas.	Food accessibility decreases through overcrowding in urban areas, where growing poverty exacerbates the situation.	Malnourishment compounded with poor water quality, sanitation led to an increase of children under 5 mortality rate- anemia, vitamin A and iodine deficiency.	-	Introduction of a food rationing system by the United Nations for all Iraqi residents in August 1990 to provide the essential staples.	Medium
Saleh, 2015^16,17^	Comprehensive	Existing macro-economic data	All Iraqi population.	The calories provided per person decreased from 3200 to 2450 calories/day.	Deteriorating purchasing power of individuals.	-	-	Adopting food ration system in the oil-for-food program; Fixing the price of food baskets.	Medium
Woertz, 2019^18^	UN/1991-2003 Oil-for-Food Program (1996)	Document analysis	Iraqi archival resources and newspapers.	Electric cuts interrupted irrigation pumps and diversion of water supplies to farmers. Reduced incentives increasingly weighed on agriculture. Input price inflation and the declining terms of trade of agriculture.	-	-	-	Prioritization of agricultural self-sufficiency especially cereal production to break the embargo used food rationing to avert famine, and instrumentalized food trade to reward cronies and punish opponents; Public distribution system that procures and allocates food at subsidized controlled prices.	Medium

Abbreviations: GDP, gross domestic product; UN, United Nations; NR, not reported.

**Table 3:** Impact of Economic Sanctions on Food and Nutrition Security and Policies to Cope With Them in Russia Based on Included Studies in the Review

**Author, Date**	**Sanction Type/Years**	**Study Design**	**Population, Sampling, Sample Size**	**Impact of Sanctions on Food Security**				**Strategies to Cope**	**Quality of the Study**
				**Availability**	**Access**	**Utilization**	**Stability**		
Kulikov et al, 2019^19,20^	Sanctions and the counter-embargo/2014	Monographic, statistical and economic methods	Existing data	Decrease in imports, reduction of investments in agricultural development.	Reduce access to vegetables, fruits, the cattle meat, and dairy products.	Low consumption of milk, vegetables, and fruits; High consumption of bread, potatoes, and sugar.	Threaten the loss of food independence, adverse climatic changes.	Growth in agricultural and food production, Import substitution Improvement of state support, the concentration of production in agricultural organizations and farmer households, development of related industries, organization of agricultural products storage, processing in the places of production, the creation of consumer cooperatives.	Medium
Volchkova and Kuznetsova, 2019^21^	Sectoral sanctions/2014	Results of the consequences of counter-sanctions	Existing data	Success of import substitution (tomatoes, pork, poultry and beef), failure of import substitution (apples, cheese, fish, condensed milk, and processed meat), very expensive import substitution (sour milk, milk and butter).	Decrease in prices and a significant increase in consumption (poultry, pork, tomatoes).		Increase in food expenditure.	Support domestic producers via trade restrictions (import substitution).	Medium
Olgarenko et al, 2019^22^	Food threats/2014	Calculation of the import substitution’s composite index	Federal districts	Inability to use scientific and technical achievements to improve the efficiency and competitiveness of their products to modernize the production.	Poor social infrastructure, Financial and economic threats.		Low productivity.	Import substitution of food for the Russian Federation in the context of the country's food security, socio-economic development, self-sufficiency in food by the regions.	Medium
Boldyreva and Rudash, 2019^23^	Economic sanctions/2014	Market-oriented economy	Existing data	The ability of the agricultural sector to ensure food processing of agricultural raw materials, and the population required for the full life of food products.	Complete self-sufficiency (cereals, vegetable oil, eggs, sugar), reaching soon complete self-sufficiency (meat of pigs and poultry, field vegetables), self-sufficiency in the long term (cattle meat, milk, vegetables of the protected ground).			The customs policy of the state includes export subsidies, tariff, and non-tariff measures to protect the domestic food market from imports.	Medium
Voronin et al, 2018^24^	Trade restrictions/2014	Economic forecasting	Existing data	Negative impact on the economy of the agrarian sector, slowing down the rate of economic growth, international trade turnover decreased.	The number of population with incomes below the subsistence minimum increased, the poverty level exceeded several points, a rise in the price of many food products.	A decrease in the level and quality of life of the population.	-	The development of agriculture, provides the population with staple foodstuffs in accordance with rational norms and medical indicators and with minimal imports of agricultural products.	Medium
Zhiryaeva, 2017^25^	Foodstuff imported/2014	Existing data	Russian citizens	Growth in agricultural production, decline in imports.	Consumer prices rose due to reduced economic availability of meat and milk.	Import substitution necessarily led to not competitive production and a decline in the quality of food.	-	Support producers, import-substitution, development of the selection and genetic engineering, development of the wholesale and distribution centers, development of a financial credit system, protect consumers.	Medium
Wengle, 2015^26^	Food embargo/2014	-	-	A significant devaluation of the ruble; the ability of Russian companies to substitute their products for imported food.	A rise in food inflation; a drop in the real income of consumers; a jump in the number of people living in poverty; the purchase of cheaper products by consumers.	-	-	The Russian strategy for achieving food self-sufficiency: (1) increase domestic production by super farms; (2) reduce food imports through the use of tariffs and non-tariff barriers.	Medium

**Table 4:** Impact of Economic Sanctions on Food and Nutrition Security and Policies to Cope With Them in Cuba Based on Included Studies in the Review

**Author, Date**	**Sanction Type/Years**	**Study Design**	**Population, Sampling, Sample Size**	**Impact of Sanctions on Food Security**				**Strategies to Cope**	**Quality of the Study**
				**Availability**	**Access**	**Utilization**	**Stability**		
Kuntz, 1994^27^	Trade — mostly in food and medicines — by US companies/1992	Investigate the current health situation in the country	The American Public Health Association members.	A 'tax' of 30% on all imports which must be purchased from markets.	The dramatic increase in the price of staple goods.	The percentage of low weight births rose 23%, mortality among Cuban women rose rapidly, women with inadequate weight gains during pregnancy or with anemia rose rapidly.	The general standard of living in Cuba and the quality of health services have declined dramatically.	Extraordinary efforts to provide extra food rations to pregnant women and retool birthing procedures.	High
Garfield and Santana, 1997^28^	All US subsidiary trade, including trade in food and medicines/1989, Tightening in 1992	Existing data and interviews	Data from surveillance systems for nutrition, supplemented with utilization data from the national health system and interviews with health leaders.	60% decline in Cuba's GDP, Importation of foodstuffs declined by about 50%.	Per capita protein and calorie availability declined by 25% and 18%, respectively, the high proportion of calories from refined sugar, which increased from 18% to 26%.	Under-nutrition is the major risk factor associated with an epidemic of optic neuropathy, the massive decline in available resources, a deteriorating public health infrastructure, and rising incidence rates for infectious diseases and low birth weight.	Health goods can no longer best retched to meet the needs of the entire population. Preferential access to essential goods for women and children has resulted in the creation of new vulnerable groups (adult men and the elderly).	Only about 1200 calories are available from low-cost rationed distribution. The entire population has been provided with monthly vitamin supplements.	High
Chaplowe, 1998^29^	The tightening of the US economic embargo in 1992	Questionnaire surveys, in addition to formal and informal interviews	Gardeners, the community members, officials, and workers from the MINAGRI, members of non-governmental organizations, Cuban Institute of Geography and faculty from the Department of Geography at the University of Havana.	The decline in food production and imports.	-	-	Shortage of petroleum needed to transport, refrigerate and store.	The government added over 200 consumer goods to the list of rationed items and reduced the quotas of two-thirds of all rationed items. Decentralizing food production and distribution (Home gardens).	Medium
Garfield, 1999^1^	Trade, 1992	Review	Available data from Cuba’s population.	Importation of foodstuffs declined by about 50%, milk production declined by 55%, average calorie availability (1992-1996): 3100 to 1865 kcal.		Poor nutrition and deteriorating sanitary conditions.		The dual policies of equity and priority for vulnerable groups.	Medium
Warwick, 1999^30,31^	Tightened existing trade embargo prevents any American from selling food or medicine to Cuba/1992	-	-	Stifle the regrowth of the Cuban economy, by deterring foreign investment.	-	-	Reducing transportation, refrigeration, and storage costs by relocating agricultural production.	Urban agriculture, organic foods.	Low
Pérez R, 2009^32^	NR	Policy and Practice	NR	Per capita daily energy intake decreased to 1863 kcal.	The FAO price index rose 28%.	Micronutrient deficiencies were associated with a neuropathy epidemic.	-	Many food staples have been sold in limited quantities to all Cuban families at subsidized prices. Land use and marketing policies are aimed at correcting insufficient, overpriced, and inconsistent supplies of fruits and vegetables and at increasing domestically-produced foodstuffs to substitute for costly imports.	Low
Rosset et al, 2011^33^	The US trade embargo	Evaluation study	Food production initially collapsed due to the loss of imported fertilizer, pesticides, tractors, parts, petroleum.	Food production initially collapsed (-5.1%) due to the loss of imported fertilizer, pesticides, tractors, parts, petroleum.	-	-	-	The ANAP used to build a grassroots agroecology movement, farming practices evolved over time and contributed to significantly increased relative and absolute production.	Medium
Drain and Barry, 2015^34^	USA Tightening the embargo/1992 Ending restrictions on selling food/2000	Review	NR	Food shortages.	-	A national epidemic of optic and peripheral neuropathy, which started was associated with malnutrition. Cuba has the highest average life expectancy, and the lowest infant and child mortality among Latin American and Caribbean countries.	-	Emphasis on disease prevention and primary healthcare. By educating their population about disease prevention and health promotion, the Cubans rely less on medical supplies to maintain a healthy population. A healthcare infrastructure to support primary-care medicine.	Medium

Abbreviations: GDP, gross domestic product; ANAP, National Association of Small Farmers; FAO, Food and Agriculture Organization; MINAGRI, Ministry of Agriculture; NR, not reported.

**Table 5:** Impact of Economic Sanctions on Food and Nutrition Security and Policies to Cope With Them in Iran Based on Included Studies in the Review

**Author, Date**	**Sanction Type/Years**	**Study Design**	**Population, Sampling, Sample Size**	**Impact of Sanctions on Food Security**				**Strategies to Cope**	**Quality of the Study**
				**Availability**	**Access**	**Utilization**	**Stability**		
Hejazi and Emamgholipour, 2022^35^	Imposition of US sanctions against Iran in 2018	ITS analysis	The average retail price of food products in Iran.	-	The percentage of urban and rural households in Iran that were prone to food insecurity increased from 8.84% and 25.17% to 11.2% and 29.2%, respectively, from 2017 to 2019.	The annual average cost of a healthy diet for a sample Iranian family of 3.3, based on the current prices is 341 866 008 IRR (US$ 2849) which is 3.6 times greater than the average amount of Iranian families spent on food last year (94 505 000 IRR or US$ 788).	A significant increase in the prices of all food groups occurred in 2018. The highest inflation rate was observed in the vegetable, meat, and fruit groups.	Creating efficient food assistance programs by the government and the international community, founding food banks with the assistance of charities and non-governmental organizations, and participation of individuals in nutritional education programs and learning how to plan a cheap and balanced diet.	High
Rustamovich, 2020^36^	Increased economic sanctions in 2012			In 2014, Iran’s GDP fell significantly from $ 6376 to $ 5293; as the result of sanctions affecting the energy, banking, and financial sectors, since.	Inflation in Iran increased from 10.7% in 2009/2010 to 39.266% in 2013/2014, resulting in more people living below the poverty line.	-	High levels of unemployment.		Low
Aloosh et al, 2019^37^	Economic sanction/2012-2015	Narrative review	Data from the World Bank and the Central Bank of Iran.	11.8% reduction in GDP growth in 2012 compared to 2011 and 14.1% increase in GDP growth in 2016 (from -1.6% to 12.5%); 40% inflation; 200% depreciation of Iranian currency; The Gini coefficient has increased from 37% to 41%.	1. More than 40% of Iran's 82 million population are living below the poverty line. 2. Unemployment increase from 10.4% in 2013 to 13.1% in 2017.	-	-	1. Active labor market programs; 2. Family support programs; 3. Provision of quality and equitable access to primary care and medications for vulnerable populations; 4. Debt relief programs.	Medium
Heidary, 2018^38^	US, Security Council and European countries sanctions/2002-2017	Longitudinal prospective study	Urban and rural households of Iran.	Calorie supply has increased from 3094 in 2010 to 3172 in 2017. The total food security score in 2010 and 2017 was 86% and 85% in the urban and 78% and 83% in the rural areas, respectively.	-	At least 10%-30% of urban households and 10%-20% of rural households received less than 2300 calories.	-	Cash transfer to reduce household inequality; Offer direct and indirect supportive packages for households; Supporting domestic production.	High
Kokabisaghi, 2018^39^	Economic sanction/2012-2017	Systematic review	55 papers.	GDP per capita decreased by 35%; GDP per capita PPP decreased by more than 10% and the value of the national currency declined by 80%.	The consumer price index increased from 100 to 178; The inflation rate increased from 20% to 38%; Minimum wage decreased from US$ 275.4 to US$ 155; Unemployment rate decreased from 10.5 to 11.3; About 11% of Iranians were living below the absolute poverty line and 30% under the relative poverty line in 2016.	-	-	-	High
Deputy Minister of Social Welfare, Ministry of Cooperatives, Labor and Social Welfare, 2018^40^	Economic sanctions and the US withdrawal from the UN Security Council/ from 2011 to 2018		-	-	6% increase in the poverty rate from 2011 to 2013; 10% increase in the poverty rate in rural areas and close to 3% in urban areas.	7% reduction in per capita expenditures of Iranian households from 2011 to 2013; Percentage of households with food poverty from 5.35 in 2011 to 9.08 in 2016; Increasing the number of households suffering from food, energy, or protein deficiency.	-	Minimum income guarantee program (to individuals who earn less than 50% of the approved minimum salary); Predicting the pensions of households covered by support institutions with a ratio of the minimum wage; Development of public employment; Integration of the social protection system.	Medium
Toghyani and Derakhshan, 2015^41^	Economic sanctions/ 1978 to 2013		Time series data.	In long term, weak and strong sanctions had no significant impact on economic growth, but moderate sanctions with coefficient 0.024 have had a Negative impact on economic growth.	-	-	-	- Managing public opinion and insisting more on the principles and maintaining the independence of the country - Activating the domestic production capacity, strategic trade policy and seriously pursuing the approach of resistance economy - Active policy-making and diversification of the drawer exchange system - Identifying and planning to support the vulnerable groups.	Medium
Ziaei et al, 2013^42,43^	Economic sanction	Cross-sectional	267 Rural households of Gorgan selected by stratified random sampling.	-	Income, employment of household head, chronic illness of one of the household members, education level of household head and spouse, household size, and access to credit had a significant effect on the nonfood coping strategy.	The mean of the coping strategy index was 15.52.	-	Most households use a coping strategy of "using less preferred and cheaper food."	Medium

Abbreviations: GDP, gross domestic product; PPP, purchasing power parity; ITS, Interrupted time series.

###  Haiti

 The embargo on Haitian exports was instituted by the Organization of American States in 1991 and partially lifted by the United States in February 1992.^8^ Initial sanctions froze the Haiti government’s assets in the United States and subsequent sanctions included more bans on imports and exports (excluding humanitarian goods). Sanctions were only lifted in 1994 after a re-establishment of the elected government.^1^

 In Haiti, the price of basic foodstuffs rose fivefold from 1991 to 1993, unemployment increased quickly, and the export of mangoes decreased, on which many poor people depended. The national currency depreciated sharply and inflation hit hard. The estimated rate of low birth weight increased from 10% to 15% of infants. In addition, 7.8% of children under the age of five suffer from acute malnutrition compared to 3.4% in 1990. One of the important factors in increasing malnutrition was the absence of the mother at home due to economic activity. This has led to less attention being paid to breastfeeding, weaning foods for young children, and caring for sick children. During the sanctions, non-governmental organizations and governments performed key tasks outside government structures to prevent the legitimacy of the military regime. Many good employees left the government at that time and never returned. This has left Haiti with a weak infrastructure.^1^

###  Iraq

 Sanctions on all items imported into Iraq except drugs began on August 6, 1990. Following the Gulf War in January and February 1991, sanctions were reaffirmed by the United Nations, and Iraq was allowed to import food in addition to medicine. Humanitarian organizations imported only 5% of the medicines and foodstuffs they deemed necessary for Iraq because their demands were not met by hostile governments. During these years, access to food to buy in the country has decreased and the power of poverty and reduced nutrition has increased and children under 2 years old are most affected. Due to the destruction of many infrastructures and the unemployment of a large number of people, despite the lifting of sanctions, many of them still depend on imports and rations produced by the government to make a living.^5^

###  Russia

 At the beginning of 2014, Russia was embargoed and had to retaliate and did not allow imports of meat and dairy products, vegetables, fruits, and fish from the United States, Canada, Australia, Norway, and the European Union states. Despite the increase in the growth of domestic production of agricultural products, its effect on food availability was offset by a decrease in imports following the introduction of general and specific commodity sanctions. The price of red meat and milk for consumers increased due to reduced economic access, and consequently, food insecurity increased from 2013 to 2015. To counter the effects of sanctions on the nutritional status of the Russian people, the Russian Food Security Doctrine was established as a general framework for agricultural policy in which minimum targets for the domestic production of products such as potatoes, dairy products, cereals, and meat were established.^25^ The impact of Russian sanctions and stop trading on the economy, finance, and food availability of other countries has also been reported in some articles^44,45^ which were not considered as the aim of the present review and so they were excluded.

###  Cuba

 The tightening of the US economic embargo in 1992 may have an unintended but profound effect on the health and nutrition of vulnerable populations in Cuba.^46^ The high prevalence of optic neuropathy from 1991 to mid-1993 in Cuba, has been attributed to an interaction of some toxins, in combination with nutritional deficiency, too.^47-51^

 Cuba’s focus on high-quality training, universal access to care, heightened vigilance for breakdowns in the social safety net, and judicious use of scarce goods shows, once again, that remarkably good health outcomes are possible if the few medical resources are put to the best use.^28,52^

###  Iran

 Major sanctions against Iran were formed after the Islamic Revolution of 1957, after the occupation of the US Embassy in Tehran, and intensified following the challenge to Iran’s nuclear program. Iran’s nuclear program has faced various sanctions since 1996; but despite the diversity and multiplicity of sanctions, for reasons such as the gradual and permanent nature of sanctions, the existence of oil revenues, adoption of import substitution strategy, global competition, lack of full compliance of independent emerging economies with sanctions, and foreign banking, their effects have diminished.^41^

 Few studies wad studied the impact of previous rounds of sanctions on the nutritional status of Iranians. A systematic review of Kokabisaghi showed that the sanctions on Iran have lowered the ability of Iranians to access the necessities of a standard life such as nutritious food and healthcare as a result of the devaluation of the national currency, increased inflation, and unemployment. These adverse effects are more severe in poor people, patients, women, and children.^39^

 The main strategies in the agricultural sector to counter sanctions are to meet the country’s food needs, support domestic production and reduce dependence, especially in the cereal, seed, livestock, and poultry industries using research and new technologies (Unpublished data).

###  Other Countries

 Sanctions, as a tool of coercive foreign policy, have been imposed on several other nations, such as North Korea, Libya, Venezuela, Sudan, Sierra Leone, Burundi, and Malawi, the apartheid government of South Africa, and former Yugoslavia, over the past few decades. However, no study was found on the impact of sanctions on food and nutrition security that was eligible for inclusion in the current systematic review. Effects of the economic sanctions on food and nutrition security and strategies to cope with them based on the review findings were summarized in Figure.

**Figure:**
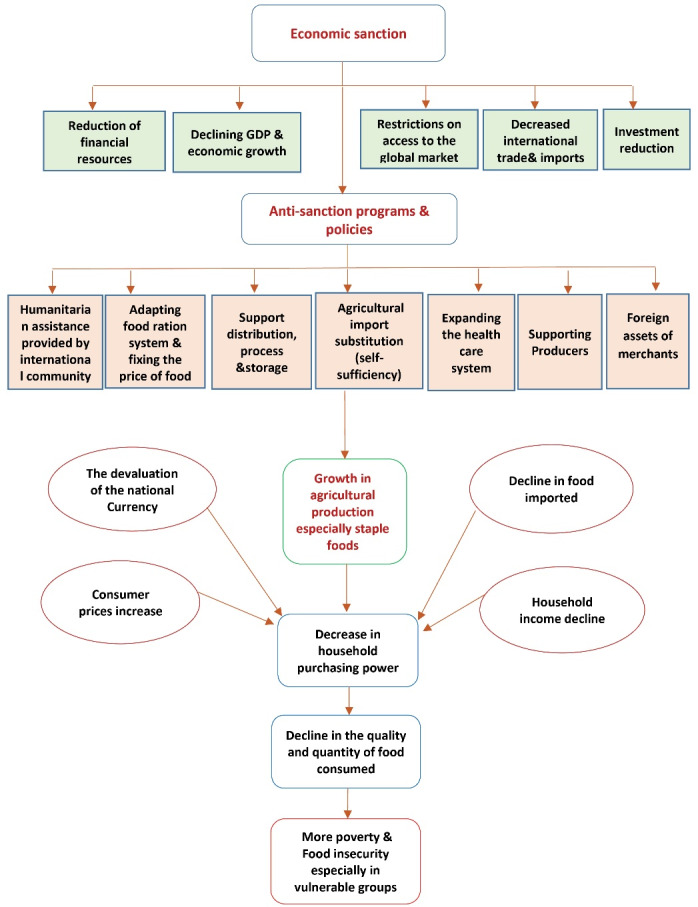
Effects of the Economic Sanctions on Food and Nutrition Security and Strategies to Cope With Them Based on the Review Findings. Abbreviation: GDP, gross domestic product.

## Discussion

 The history of sanctions returns to the First World War, in which Germany was subject to a shipping embargo by the Allied forces. As a result of the increased risk of deprivation, German children suffered severe malnutrition, and due to the blockade working-class children suffered the most.^53-55^ So, sanctions have been interpreted as a weapon ever since. The US government tried to prohibit the sale of grain to the Soviet Union before 1971 and to embargo all grain sales to the Soviets in 1999.^56,57^ More recently, the United Nations and the United States have, however, not planned for any economic sanctions on Ethiopia and Eritrea, which could compel both countries to de-escalate, because these sanctions would hurt the people of both countries which are highly dependent on food aides from international organizations.^58,59^

 Targeted regimes and their populations may experience multiple political consequences, such as weakening some regimes and strengthening others. Economic sanctions may also influence conflicts and security in targeted states. The intensity of such impacts varies even for targets, often depending on the internal dynamics of the targeted states and their relations with other countries. Sanction threats, types of sanctions, and their senders are important for the domestic politics of targets, and threats of especially multilateral sanctions increase political activity in targeted states due to their signaling effect of outside support to opposition.^60-62^

 A review by Gutmann et al found that UN sanctions reduce life expectancy by 1.2–1.4 years and US sanctions by 0.4–0.5 years. Increased child mortality and deaths from cholera, along with declining public health expenditures, have been the main reason for the decline in life expectancy. Women are heavily affected by sanctions, too. There are concerns in the world about the undue effects of economic sanctions on human rights, especially children. Economic sanctions against countries are inconsistent with the United Nations Convention on the Rights of the Child, which deals with children’s rights to access healthcare, social welfare, and education.^63^ Qualitative research based on two countries’ case studies found that sanctions negatively affect the availability of food and clean water. These findings are corroborant with quantitative ones.^2,28^ Qualitative information is essential for developing useful causal models. Key informants from the social programs or data collection agencies already have a detailed understanding of which variables are related and the nature of their influence on each other.^64^

 There is a growing policy consensus that economic sanctions are powerful tools to cope with major foreign policy crises. However, the real effectiveness of sanctions, particularly targeted sanctions and the circumstances in which policy change induce in sanctioned countries are in question.^65^ However, studies in different countries exposed to sanctions indicate deteriorating food security and nutritional status of the people particularly poor and vulnerable groups. These effects are mainly mediated by the increase in unemployment and food prices, decrease in food imports and purchasing power, and ultimately poverty increase.^39^ The impact of sanctions on poverty (*a*) increases with the severity of sanctions, (*b*) is larger for multilateral sanctions than for unilateral sanctions imposed by the United States, and (*c*) becomes longer lasting as the poverty gap widens 3.8 percentage points in sanctioned countries compared to the control group in the first 21 years of an embargo. A slowdown in exports and imports, as well as a reduction in foreign aid, are other pathways through which US sanctions negatively affect the target’s poverty level.^66^

 Analysis of household expenditure data showed that — despite efforts of the agricultural sector — production, supply, and purchasing of milk and dairy products, red meats, and fish have decreased in both Iranian urban and rural households, especially after the cash transfer program, began the sanctions and inflation. However, the consumption of bread as the main staple food and oil remained almost unchanged.^67^ World Food Program^68^ also reported the marked deterioration of the macroeconomic performance of Iran following the subsidy reform in 2010 and the intensification of sanctions in 2012. Real gross domestic product (GDP) fell by 5.8% in the year 2012/2013 and inflation increased by 41.2%. Soaring food prices and subsidy cuts have directly affected the food security situation among the poor and vulnerable population. Higher infant and under 5-year’s mortality, energy, and protein deficiency, and poverty rate are also reported.

 Due to the role of a healthy diet (high amounts of fruits and vegetables, whole grains, legumes, low-fat milk and dairy products, and seafood) in preventing non-communicable diseases, reducing milk and dairy consumption, fish, and legumes, in the long run, can lead to increased burden of non-communicable diseases such as cardiovascular disease, hypertension, diabetes, and osteoporosis in populations under sanctions.^69-71^

 Food aid can be considered as one of the strategies to reduce the effects of the embargo on the food security of embargoed countries. Despite the arguments made against the politicization of aid, mostly bilateral and multilateral aid remains tied to the political goals of rich countries. Such a link can be fragile (for example, aid is paid only if certain economic policies or political systems are adopted).^11,72^ On the other hand, the impact of sanctions on humanitarian action is closely related to the spread of counter-terrorism measures more broadly, which negatively affects the ability of humanitarian actors to operate. A restrictive environment for humanitarian actors, and their compounding effect leads to some challenges such as costs and delays caused by exemption procedures, restrictions on importing goods, restrictive clauses in donor agreements, fines, and prosecution.^73,74^

 In Haiti, relying on international aid to maintain food security was not enough; In Iraq, the main strategy was to ration and deliver the minimum required food to all households based on the oil versus food program^1^; In Cuba, reinforcement of the health services system and preventing mothers and children mortality led to the death of the elderly and men^28^; In Russia, self-reliance was possible due to climatic conditions. Iran tried a combination of coping strategies aiming to maintain a minimum of food required for the lower deciles was applied.

###  Limitations

 Most of the studies were based on the existing routine data gathered in the imposed countries^16,17^ and the use of both qualitative and quantitative literature is a positive attribute of this Review. The JBI Reviewer’s Manual guides to authors for the conduct and preparation of many kinds of systematic reviews and evidence syntheses. Therefore, considering the diversity of the types of studies reviewed and that the JBI Reviewer’s Manual included guidelines for almost all types of studies, we used JBI systematic reviews of mixed methods guidelines to quality assess the included studies. However, the JBI checklist is complex and detailed and its use for policy review is difficult which led to the assignment of low quality to many studies included in the review. The systematic review took longer than expected, because of the dispersion of studies and the COVID-19 pandemic made communication and sharing opinions difficult among the research team. Access to some scientific resources was limited, which makes it difficult to ensure access to all sources for a systematic review.

 Since at the time of this review, much time has passed since the embargo in the countries and the studies carried out on its effects (10 years or more), likely the conditions described in most of the sanctioned countries, especially for vulnerable groups have gotten worse. Furthermore, a meta-analysis does not capture much of the gray literature that captures the social context or narratives of the affected population. It is a flaw in any meta-analysis that it does not fully capture what’s going on.

## Conclusion

 Ample evidence was found about the adverse effects of any kind of economic sanctions on the welfare, health, and food security of civilians in embargoed countries, which are mainly mediated by the increase in unemployment and food prices, decrease in food imports and, purchasing power. Because the increasing rate of poverty caused by sanctions affects particularly the most disadvantaged and vulnerable groups, planning to improve food security and provide support packages for these groups (eg, women and children in low-income households) seems essential.

 The most important strategies to improve food security in countries under economic sanctions can be summarized in four categories: the humanitarian assistance provided by the international community (Haiti), equity, and priority for vulnerable groups mainly by expanding the healthcare system (Cuba), adopting food ration system in the oil-for-food program and fixing the price of food baskets (Iraq), and supporting production, processing, and distributing food to consumers (import substitution and self-sufficiency in Russia), support domestic production and reducing dependency especially in staple cereals, seeds, livestock and, poultry industry using new research and technologies, direct and indirect support and compensation packages for vulnerable households (the approach of resistance economy in Iran).

 The effectiveness of sanctions is determined only by their political outcome, and the suffering of the people of these countries due to malnutrition in addition to their social and biological consequences is ignored.

## Acknowledgements

 This systematic review was conducted in the National Nutrition and Food Technology Research Institute (NNFTRI) of Iran in the framework of the research project entitled “Study of economic sanctions affecting household food and nutrition security, policies to cope with them and offer a supportive package in Iran” (Prospero Registration No. CRD42020191028). The authors thank the research deputy of NNFTRI and all researchers who made this review possible through their valuable studies.

## Ethical issues

 This study was approved by Ethics Committee of National Nutrition and Food Technology Research Institute, Shahid Beheshti University of Medical Sciences (IR.SBMU.NNFTRI.REC.1398.053).

## Competing interests

 Authors declare that they have no competing interests.

## Funding

 This study was funded by the NNFTRI of Iran (Grant No. 99-18177).

## Supplementary files

Supplementary file 1. Search Strategy.

Supplementary file 2 contains Figure S1.
