# Supplementary material for: Economic Sanctions Affecting Household Food and Nutrition Security and Policies to Cope With Them: A Systematic Review
Source: Int J Health Policy Manag. 2023 Aug 5;12:7362. doi: 10.34172/ijhpm.2023.7362 (PMC10590471; doi:10.34172/ijhpm.2023.7362)
Supplement: Supplementary file 1 — Search Strategy. [file ijhpm-12-7362-s001.pdf]

**Article title:** Economic Sanctions Affecting Household Food and Nutrition Security and Policies to Cope With Them: A Systematic Review

**Journal name:** International Journal of Health Policy and Management (IJHPM)

**Authors' information:** Fatemeh Mohammadi-Nasrabadi[1]*, Delaram Ghodsi[2], Arezoo Haghighian-Roudsari[3], Fatemeh Esfarjani[1], Mohammad-Reza Khoshfetrat[1], Zeinab Houshialsadat[4], Maryam Mohammadi-Nasrabadi[5], Ghasem Fadavi[6]*, Reza Majdzadeh[7]

[1]Research Department of Food and Nutrition Policy and Planning, National Nutrition and Food Technology Research Institute, Faculty of Nutrition Sciences and Food Technology, Shahid Beheshti University of Medical Sciences, Tehran, Iran.

[2]Department of Nutrition Research, National Nutrition and Food Technology Research Institute, Faculty of Nutrition Sciences and Food Technology, Shahid Beheshti University of Medical Sciences, Tehran, Iran.

[3]Department of Community Nutrition, National Nutrition and Food Technology Research Institute, Faculty of Nutrition Sciences and Food Technology, Shahid Beheshti University of Medical Sciences, Tehran, Iran.

[4]School of Public Health, Physiotherapy and Sports Sciences, University College Dublin, Dublin, Ireland.

[5]Department of Health Education and Promotion, School of Public Health, Tehran University of Medical Sciences, Tehran, Iran.

[6]Food, Halal and Agricultural Products Research Group, Research Center of Food Technology and Agricultural Products, Standard Research Institute, Karaj, Iran.

[7]Interdisciplinary Research and Practice Division, School of Health and Social Care, University of Essex, Colchester, UK.

*Correspondence to: Fatemeh Mohammadi-Nasrabadi, Email: f.mohammadinasrabadi@sbmu.ac.ir & Ghasem Fadavi, Email: fadavi@standard.ac.ir

Citation: Mohammadi-Nasrabadi F, Ghodsi D, Haghighian-Roudsari A, et al. Economic sanctions affecting household food and nutrition security and policies to cope with them: a systematic review. Int J Health Policy Manag. 2023;12:7362. doi:10.34172/ijhpm.2023.7362

**Supplementary file 1.** Search Strategy

**Web of Science (187)**

**ALL FIELDS:** (("economic sanction" OR embargo)) *AND* **ALL FIELDS:** ((food* OR nutrition OR nutrient OR "food secur*" OR "food insecur*"))

**Science Direct (404)**

Title, abstract, keywords: ("economic sanction" OR embargo OR "monetary sanction") AND (food* OR nutrition OR nutrient OR "food secur*" OR "food insecur*")

**Embase (57)**

("economic sanction" OR embargo) AND (food* OR nutrition OR nutrient OR "food secur*" OR "food insecur*")

**Escopus (257)**

( TITLE-ABS-KEY ( ( "economic sanction" OR "embargo" ) ) AND TITLE-ABS-KEY ( ( food * OR nutrition OR nutrient OR "food secur*" OR "food insecur*") ) ) AND PUBYEAR > 1989

**Pubmed: 45**

((("economic sanction" OR embargo))) AND (food* OR nutrition OR nutrient OR "food secur*" OR "food insecur*")

**JSTOR: 337**

("economic sanction" OR embargo) AND (food* OR nutrition OR nutrient OR "food secur*" OR "food insecur*")

**Google scholar (56)**

**TITLE:** (("economic sanction" OR embargo OR "monetory sanction")) *AND* **TITLE:** ((food* OR nutrition OR nutrient OR "food secur*" OR "food insecur*"))
